# Comparison of a human acellular dermal matrix and a polypropylene mesh for pelvic floor reconstruction: a randomized trial study in a rabbit model

**DOI:** 10.1038/s41598-022-22190-4

**Published:** 2022-11-30

**Authors:** Marta Pero, Cristina Castells-Sala, Leticia Alserawan, Laura Casani, Josep Oriol Juan Babot, Ignasi Jorba, Maria Luisa Pérez, Esther Moga, Jorge Otero, Patricia López-Chicón, Lina Badimon, Anna Vilarrodona Serrat, Oriol Porta-Roda

**Affiliations:** 1 Department of Obstetrics and Gynecology, Hospital de La Santa Creu I Sant Pau, Biomedical Research Institute Sant Pau (IIB Sant Pau), Universitat Autònoma de Barcelona, Carrer Sant Quintí 89, 08041 Barcelona, Spain; 2 Biomedical Research Institute Sant Pau (IIB Sant Pau), Barcelona, Spain; 3 Barcelona Tissue Bank. Banc de Sang I Teixits (BST), Barcelona, Spain; 4 Department of Immunology, Hospital de La Santa Creu I Sant Pau, Biomedical Research Institute Sant Pau (IIB Sant Pau), Universitat Autònoma de Barcelona, Barcelona, Spain; 5 Vall Hebron Institute of Research (VHIR), Barcelona, Spain; 6 Department of Biomedical Engineering, Institute for Complex Molecular Systems (ICMS), Eindhoven University of Technology, Eindhoven, The Netherlands; 7 Biophysics and Bioengineering Unit, University of Barcelona, Barcelona, Spain; 8 Department of Obstetrics and Gynecology, Hospital Universitari Mútua Terrassa, Universitat de Barcelona, Barcelona, Spain; 9 Department of Immunology, Hospital Clinic de Barcelona, Barcelona, Spain

**Keywords:** Experimental models of disease, Implants, Tissues, Regenerative medicine, Tissue engineering, Sexual dysfunction, Urinary incontinence, Reconstruction, Outcomes research, Preclinical research, Translational research, Chronic inflammation

## Abstract

Non-absorbable polypropylene (PP) meshes have been widely used in surgical reconstruction of the pelvic floor disorders. However, they are associated with serious complications. Human acellular dermal matrices (hADM) have demonstrated safety and efficacy in reconstructive medicine, but their suitability and efficacy at vaginal level is not known. This study compares the biological performance of PP mesh and a newly developed hADM. 20 rabbits were randomized to receive the hADM graft or the PP mesh. Grafts were surgically implanted in the abdominal wall and vagina. After 180 days, grafts were explanted and evaluated. The vaginal mesh extrusion rate was higher in the PP group (33% vs. 0%, p = 0.015). Full integration of the vaginal grafts was more frequent in the hADM group, where 35% of the grafts were difficult to recognize. In the PP group, the vaginal mesh was identified in 100% of the animals (*p* = 0.014). In PP group, the infiltrates had a focal distribution and were mostly located in the internal part of the epithelium, while in the hADM group, the infiltrates had a diffuse distribution. Additionally, the hADM group also presented more B-lymphocytes and less T-lymphocytes. Biomechanical analysis showed that hADM had lower resistance to stress. Moreover, PP mesh stiffness and elasticity were higher. Then, hADM is associated with fewer clinical complications, as well as better tissue integration. However, it shows greater incorporation into the surrounding native tissue, especially in the vaginal location, undergoing a reduction in its biomechanical properties 6 months after implantation.

## Introduction

Pelvic floor dysfunction is a very common condition that affects 30–50% of the adult female population^1^. Surgical repair may involve the use of a non-absorbable synthetic macroporous prosthesis (polypropylene [PP] meshes). However, synthetic meshes may induce a severe chronic inflammatory response, and have been associated with severe complications such as erosion, retraction, and pain^2^. Due to the risk of complications associated with their use in pelvic organ prolapse (POP) and urinary incontincence (UI) surgery, some international regulatory authorities^3,4^ have banned vaginal mesh implants for pelvic surgeries since July 2018. Accordingly, there is a need to investigate safer and more biocompatible materials to improve the current situation with PP meshes.

Tissue engineering is emerging as a new technology for tissue regeneration when native tissues are compromised. Application of these novel approaches may overcome the problem of weakened pelvic floor that occurs in women with POP or UI and offer personalized therapy for patients. This research will help to solve an as yet unmet clinical need.

Acellular matrices (AM)—decellularized scaffolds of biological origin—have emerged as good candidates to replace synthetic meshes. In acellular dermal matrices (ADM), the architecture and components of the extracellular matrix are mostly preserved, making them useful for clinical applications where tissues need to be replaced or reinforced, such as rotator cuff repair, and breast or abdominal wall reconstructions^5–7^. Dermal allografts have demonstrated their safety and clinical efficacy in different clinical applications^8,9^, but their application in the field of gynecology has been scarcely evaluated^10–15^.

Our group has previously reported the development of an acellular dermal matrix from human cadaveric donors (hADM)^16^, an allograft with a set of mechanical, structural, biochemical and storage properties suitable for a broad range of clinical applications, including gynecology.

We have also previously reported the development of an animal model to study PP and hADM grafting in the vaginal position^17^, showing that the New Zealand White (NZW) rabbit is a suitable model for assessing engrafting of biomaterials in vaginal and pelvic reconstructive surgery. The hADM developed was associated with fewer clinical complications, as well as better macroscopic tissue integration than PP meshes.

Therefore, the aim of the present study was to fully characterize the biological properties of hADM, its integration capacity and response in an in vivo animal study, in order to determine the safety and potential use of this graft in pelvic floor reconstructive surgeries.

## Materials and methods

### Preparation of hADM samples

The study was performed according to European and Spanish Directives for Tissues and Cells for tissue donation, retrieval, processing and preservation and clinical use.

Ethics institutional review board (IRB) approval was obtained (CEIm Hospital Valle Hebrón, Barcelona; PR (BST)314/2019) for human cadaveric donors. Also research purposes of the tissue was obtained through signed informed consent. No tissues were obtained from the prisoners.

The skin decellularization protocol was previously defined to remove the cellular content while maintaining the structure and mechanical properties of the native tissue. 16 Skin fragments were decontaminated and decellularized by hypertonic and hypotonic solutions, followed by an enzymatic treatment and a detergent-soaking step. Finally, hADM fragments were trimmed to 20 × 10 mm and preserved in a glycerol solution.

### Animal model

Experimental procedures were reviewed and approved by the Institutional Animal Care and Use Committee of the Research Institute at the Hospital de la Santa Creu i Sant Pau and authorized by the Animal Experimental Committee of the local government authority (Generalitat de Catalunya, authorization No. 9669) in accordance to the Spanish law (RD 53/2013) and European Directive 2010/63/EU. In addition, the investigation conforms to the Guide for the Care and Use of Laboratory Animals published by the US National Institutes of Health (NIH Publication No. 85–23, revised 1985), follows the ARRIVE guidelines (Animal Research: Reporting of In Vivo Experiments), and is committed to the 3Rs of laboratory animal research and consequently used the minimal number of animals to reach statistical significance.

An in vivo study based on a NZW rabbit model was previously performed to evaluate its feasibility^17^. hADM was compared to PP mesh in abdominal and vaginal locations. Briefly, a total of 20 female multiparous NZW rabbits were randomly assigned to receive a PP mesh (control group) or hADM graft (experimental group). Each rabbit received four grafts: two grafts in the vaginal submucosa layer and two grafts in the subcutaneous tissue of the abdominal wall. For the vaginal implants, one graft (5 × 5 mm) was placed in the anterior vaginal wall and was used for histological and immunohistochemical studies. The second graft (10 × 5 mm) was placed in the submucosa of the posterior vaginal wall and was used to perform the biomechanical study. Both abdominal grafts were implanted in the right caudal quarter of the abdominal wall. The surgical procedure and animal housing were performed following the protocol previously published by Pero et al.^17^.

### Explant retrieval

After 180 days of veterinarian monitoring, the rabbits were euthanized and the grafts explanted, removing the surrounding tissue from the prosthesis (explants).

### Histological study

Explants (5 × 5 mm) from the abdomen and vagina were immersed in fixative solution (4% paraformaldehyde) and embedded in paraffin with an automatic tissue processer (Thermo Shandon, Citadel 2000). Paraffin blocks were cut into 5 μm-thick serial sections with a microtome (Leica, JUNG RM2055) and placed on poly-L-lysine coated glass slides. Histological slides were observed using a binocular transmitted light microscope (Nikon Eclipse 80i) at 8X, 40X and 100X magnifications. Images were acquired using a digital camera (R3 Retiga, Qimaging) and processed with the software Image-Pro 10 (Media Cybernetics). Morphological, histological and immunohistochemical properties were analyzed.

The following staining was performed: hematoxylin–eosin (H&E) to evaluate the structure and grade of inflammatory reaction, Masson's Trichrome to evaluate the collagen fiber organization, and Sirius Red to evaluate the organization of collagen fibers (type I and III) of the explants. A macroscopic image at 100X magnification of each explant was performed (*n* = 10 hADM; *n* = 9 PP) and thereafter the images were quantified using ImageJ software (Launcher). The percentage of fibrosis was calculated as the stained area (SA) with respect to the total area (TA): (SA/TA)*100.

### Immunohistochemical study

Anti-CD3 (T-cell marker), anti-CD79a (B-cell marker) and anti-RAM11 (macrophage marker) antibodies were used to assess the cellular inflammatory profile in the sections with cellular infiltrates observed in the H&E staining. Sections were deparaffined and the antigen was recovered. The sections were washed with phosphate buffered saline (PBS) and incubated with 5% hydrogen peroxide for 30 min. Subsequently, 1 h of blocking was performed using a blocking solution composed of 0.2% albumin, 1% mouse serum and 0.5% goat serum. The sections were then incubated for 1 h with mouse primary antibodies against CD3 markers (CD3 epsilon antibody PC3/188A, Novusbio), CD79a (CD79a HM57, Bio-Rad) and macrophages (RAM11, Dako). This was followed by incubation with peroxidase-conjugated goat anti-mouse secondary antibody (Jackson ImmunoResearch). Finally, the reaction was revealed by incubation with the DAB substrate (Vector Laboratories) for 10 min and hematoxylin counterstaining. The slides were fixed and dehydrated on an ethanol-xylene gradient and covered with Entellan® mounting medium.

The presence of macrophages, B cells and T cells present in the inflammatory infiltrates (100X magnification) was interpreted using a 0 to 4 + scale: 0 indicates absence of cells; 1 + indicates scattered presence of isolated cells; 2 + indicates slight presence; 3 + indicates moderate presence; and 4 + indicates severe presence. The results were subsequently grouped into 2 categories: mild presence (1 + , 2 +) and severe presence (3 + , 4 +).

### Biomechanical assay

**Figure 1:**
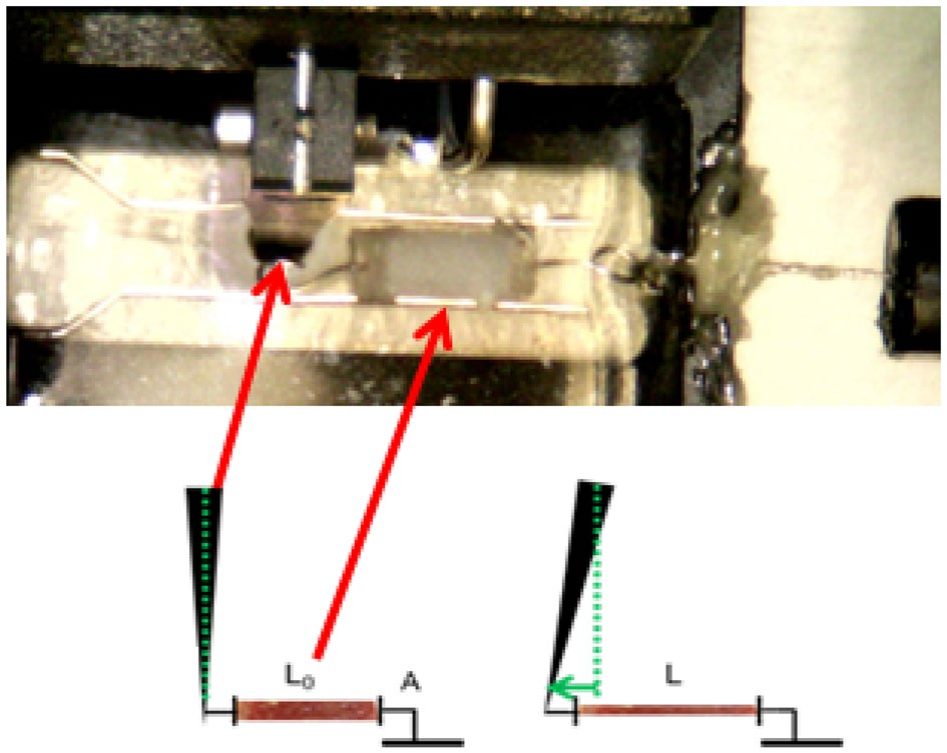
Photograph and diagram of the experimental setup for biomechanical testing of the implants.

Biomechanical properties were analyzed using the uniaxial tensile stretch test. The analyzed samples were native human skin and baseline hADM (not implanted sample), and the explanted grafts (both hADM and PP mesh). Each sample of native human skin, baseline hADM, or explanted hADM was cut into three different strips (replicates) of ~ 5 mm × 1 mm × 0.7 mm. For each strip, one end was attached with cyanoacrylate glue to a fixed hook while the other was glued to a hook attached to the lever of a servo-controlled displacement actuator with an integrated 1 N force sensor (300C-LR, Aurora Scientific, Aurora, Canada) (see Fig. 1). In the case of PP explanted meshes, the complete fragment was measured using a 5 N force sensor (305C, Aurora Scientific, Aurora, Canada). The stress–strain (*σ-ε)* curves were calculated from the force–displacement curves using geometrical measurements of the sample made by a calibrated CCD camera. Biomechanics of the samples were characterized as the stress measured at the 20% stretch point^18^.

### Statistical analysis

A descriptive analysis was performed, determining the median and also the mean with standard deviation (SD). The relationship between categorical variables was analyzed using the corresponding contingency tables, calculating the percentage in each group and applying the chi-square test with approximation of the probability ratio. For the ordinal variables, the comparison between two groups was made using the non-parametric Mann–Whitney test. In all cases, the usual level of significance was 5% (alpha = 0.05). In the case of the biomechanical study, the Student t-test was performed to compare differences in *σ* and *E*_*M*_ in the different groups at *ε* = 0.2. All analyses were performed with the statistical package IBM-SPSS (V25).

## Results

### Preparation of hADM samples

Twenty-five pieces of hADM measuring 20 × 10 mm were prepared. Each decellularized piece had a low DNA content, specifically 0.55 ± 0.68 ng/mg dry tissue and 1.03 ± 0.71 ng/mg dry tissue, while maintaining the extracellular matrix (ECM) structure and major proteins^16^.

### Explant retrieval

Twenty animals were included, ten in the experimental group (hADM) and ten in the control group (PP mesh). One rabbit from the control group died due to causes unrelated to the surgeries or graft. After six months of housing in the animal facilities, both abdominal and vaginal implants were explanted. All PP meshes in both the abdominal and vaginal location (*n* = 36) and all hADM in the abdominal location (*n* = 20) were recovered. However, only 13 of the vaginal hADM implants (65%) were recovered; the other seven matrices were integrated in the rabbit vaginal mucosa and could not be explanted.

### Macroscopic study

Table 1 describes the pathological findings observed during explantation surgery. PP implants showed mesh extrusion at both the vaginal (33%; *p* < 0.05) and abdominal (11%) location, compared to hADM implants (0% mesh extrusion). The PP group presented a higher incidence of chronic infection such as abscess formation and wound dehiscence than the hADM group. Chronic infection was 33% vs. 10% in the abdominal position and 11% versus 10% in the vaginal position. All PP meshes were recovered from both the vaginal and abdominal sites. Of ten hADM vaginally implanted, six were fully integrated with the surrounding tissue, but two were able to be identified through the non-absorbable prolene stitches used to attach them to the host during the implant surgery. Therefore, six hADM were recovered and further analyzed.

**Table 1:** Macroscopic study of explants.

	PP mesh (*n* = 9, *N* = 36)	hADM (*n* = 10, *N* = 26)	*p* value*
Vaginal mesh extrusion	3 (33%)	0	0.024
Abdominal mesh extrusion	1 (11%)	0	0.474
Chronic infection signs in abdomen location	3 (33%)	1 (10%)	0.303
Chronic infection signs in vaginal location	1 (11%)	1 (10%)	1
Vaginal graft recovered	9 (100%)	6 (60%)	0.014

*N* number of animals explanted, *n* number of analyzed samples.

*p < 0.05.

### Histological study

**Figure 2:**
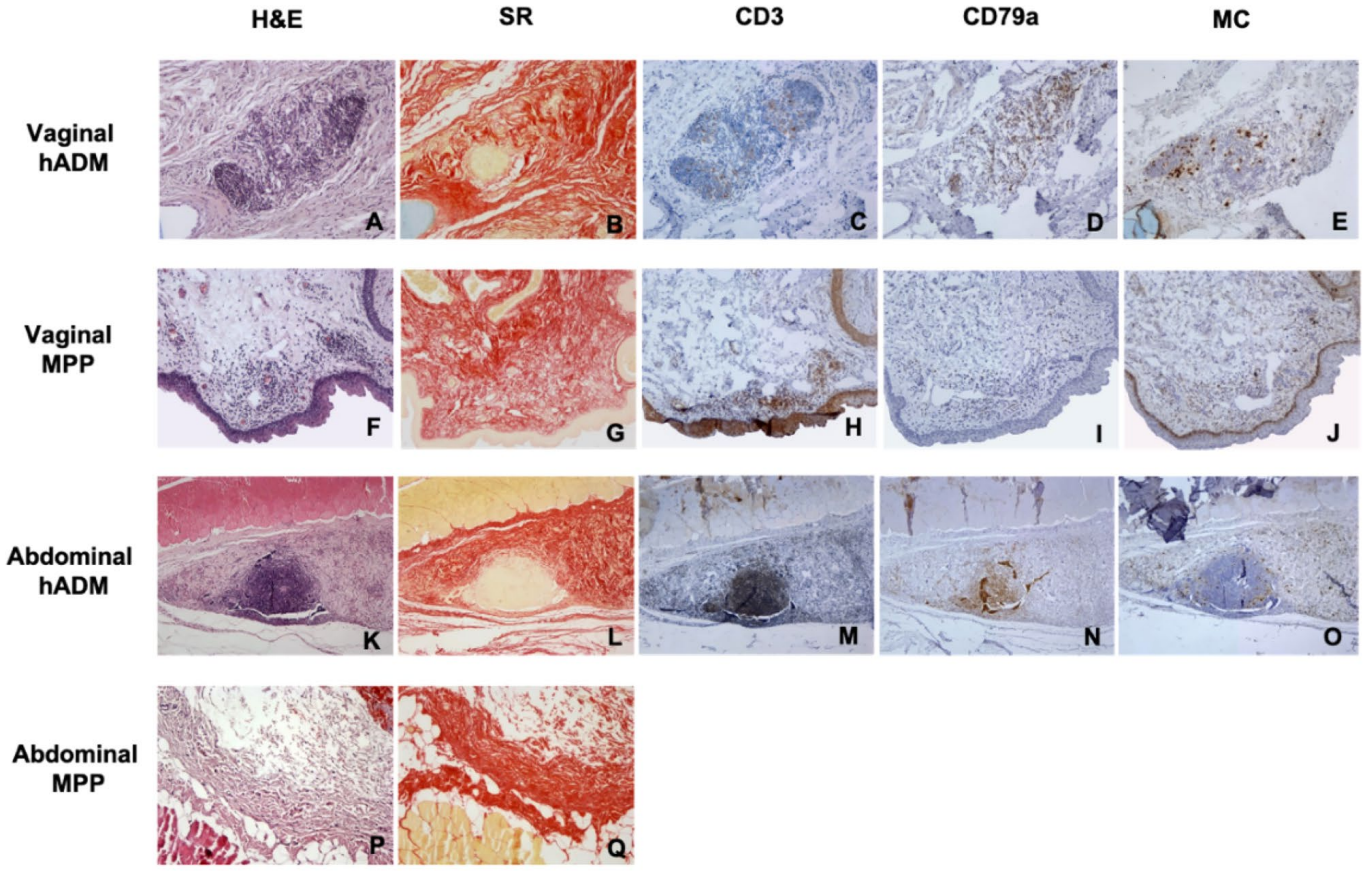
Representative histology and immunohistochemical staining of serial vaginal (**A**–**J**) and abdominal (**K**–**Q**) sections: Hematoxylin–Eosin (**H**&**E**), (**A**,** F**,** K**,** P**), Sirius Red (SR), (**B**,**G**,**L**,**Q**), T lymphocytes (CD3), (C,H,M), B lymphocytes (CD79a), (**D**,**I**,**N**) and macrophages (MC), (**E**,**J**,**O**), (X100).

Eighteen PP explanted meshes and eighteen explanted hADM samples were analyzed both histologically and immunohistochemically. The H&E study (Table 2) showed that 4/9 animals (44%) treated with PP mesh (control group) presented inflammatory infiltrates in the vaginal explants. These infiltrates had a predominantly focal distribution and were located in the internal part of the epithelium (Fig. 2F,G,H,I,J). No animal treated with the PP mesh presented inflammatory infiltrates at the abdominal level (Fig. 2P,Q). Additionally, 100% of the animals treated with the hADM presented inflammatory infiltrates. A large number of foci of infiltrates were identified at both the vaginal and abdominal sites (compared with the control group, showing diffuse distribution of the infiltrates, Figs. 2A,B,C,D,E,K,L,M,N,O, respectively). One animal showed infiltrates in both the vaginal and abdominal positions.

**Table 2:** Number of inflammatory infiltrates observed. Statistical analysis was performed using chi-square test.

	PP mesh (*n* = 9)	hADM (*n* = 9)	*p* value
Infiltrates	4	9	0.040
Vagina	4	5	0.630
Abdomen	0	5	0.008

*n* = number of analyzed samples.

Sirius Red staining showed the percentage of fibrosis of each type of explant and location (Figs. 2B,G,L,Q). The percentage of fibrosis was higher in the control group. The arrangement of collagen fibers was more compact in this group, especially in the abdominal location (Fig. 2Q). Unlike in the experimental group, the collagen structure remained stable (Figs. 2B,L).

### Immunohistochemical study

Immunohistochemical analysis (Table 3) showed that in the experimental group, 7/10 infiltrates (70%) had a slight T lymphocyte (TL) expression and high macrophage expression. The presence of B lymphocytes (BL) depended on the location, with high expression being observed at the vaginal level and mild expression at the abdominal level. Regarding the control group, the presence of BL was low in all cases, variable in macrophages and high in TL. No animal in the control group presented infiltrates in the abdomen.

**Table 3:** Presence of CD3 + cells, CD79 + cells and macrophages in the hADM group and PP mesh group biopsies. Cellular presence was quantified using a 0–4 + scale and these data were grouped into 2 categories: mild (1 + , 2 +) and severe (3 + , 4 +).

		hADM		PP mesh	
		Vaginal (*n* = 5)	Abdominal (*n* = 5)	Vaginal (*n* = 4)	Abdominal (*n* = 0)
CD3 (TL)	MP	4	3	1	0
	SP	1	2	3	0
CD79a (BL)	MP	0	4	4	0
	SP	4	1	0	0
Macrophages	MP	2	2	1	0
	SP	3	3	2	0

*MP* mild presence; *SP* severe presence.

*n* = number of analyzed samples.

### Biomechanical assay

A total of ten samples of native human skin, ten samples of baseline hADM, sixteen PP explanted meshes (nine from the abdominal area and seven from the vaginal area) and twelve explanted hADM samples (eight from the abdominal area and four from the vaginal area) were measured, as detailed in Table 4.

**Table 4:** Description of the analyzed explants during the mechanical test.

Location	PP mesh (*n* = 9)	hADM (*n* = 10)
Vagina	7	4
Abdominal wall	9	8

*n* = number of analyzed samples.

Three vaginal hADM explants could not be identified due to their integration into the host tissue, while three vaginal hADM explants were not measured due to difficulties during sample dissection. The hADM placed in the abdominal position were clearly identified, but two of them were not measured due to technical problems during sample preparation for the experiment. With regards to PP meshes, two vaginal samples with PP explants could not be measured due to similar technical problems. One PP mesh subject died during the follow-up.

**Figure 3:**
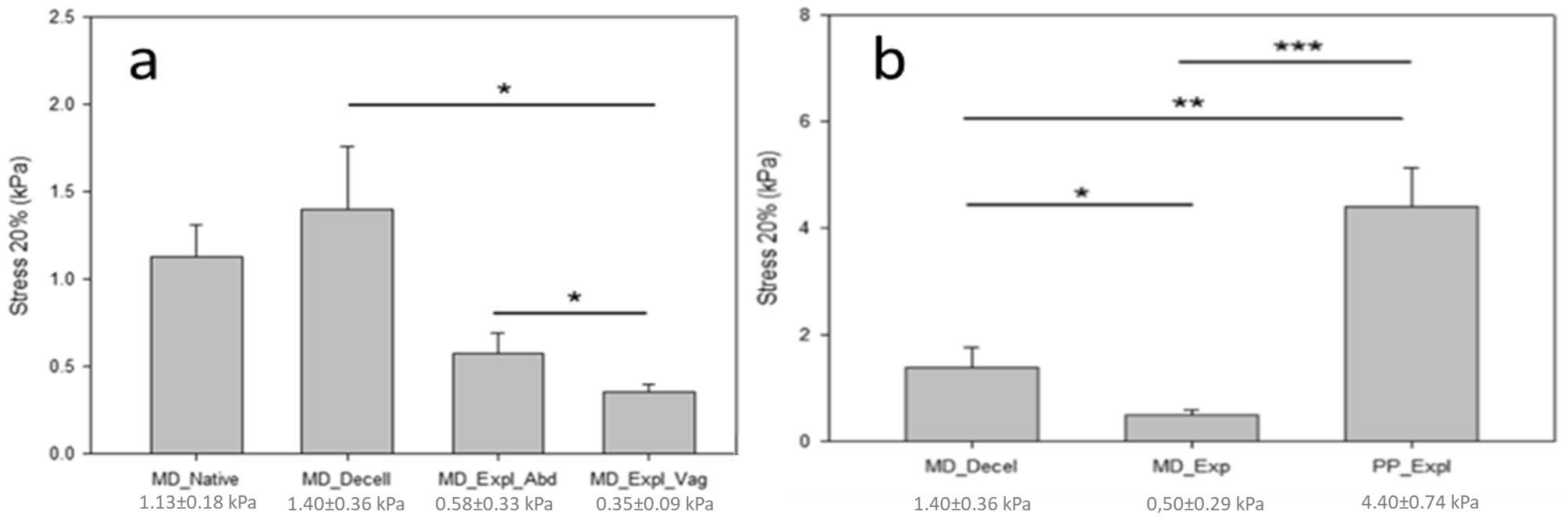
(**a**) Mechanical properties of the hADM grafts before and after their implantation. (**b**) Comparison of mechanical properties between explanted hADM and explanted PP mesh in the abdominal location. *p value < 0.05, **p value < 0.01, ***p value < 0.001.

Results in Fig. 3 show that the stress at 20% of stretch of the explants in the experimental group decreased, especially at the vaginal location, compared to the pre-implanted biomechanical properties. PP mesh explants were found to have a higher stiffness compared with hADM explants. Explanted hADM showed significantly lower stiffness than hADM prior to the in vivo implantation.

No differences were observed between the stiffness of PP meshes explanted from the vaginal or the abdominal zone. However, a significant decrease in the stiffness of hADM explants from the vagina was observed compared to the stiffness of the hADM explants from the abdominal area (*p* < 0.05).

## Discussion

The most important finding of this study was that human dermal matrices implanted in both the abdominal and vaginal site in rabbits were safe. The complete histological characterization of the explants confirmed that new tissue was regenerated in the vaginal site, with maintenance of a well-organized collagen fiber structure that supported the repopulation.

The macroscopic findings of the explants showed very different behavior between the two groups, and these differences were especially relevant in the vaginal location. The control (PP) group showed a statistically significant increase in vaginal extrusion, compared to hADM. These results were expected, since vaginal extrusion of PP meshes is a known common complication. On the other hand, the hADM graft was completely integrated into the surrounding tissue in a significant percentage of cases, and in some subjects, it was impossible to identify and remove the explants, especially at the vaginal level. These results are consistent with previous publications showing 70% and 100% graft degradation in the vaginal location by Pierce et al. and Claerhout et al., respectively^19,20^. The macroscopic findings in our study are therefore consistent with excellent biocompatibility of hADM grafts at the vaginal level.

The inflammatory response is a physiological process that occurs as a consequence of the immunological interaction with a foreign body. Nevertheless, the nature and intensity of this response depend on the characteristics of the implanted material. The results of this study reveal that the immune responses are different in both groups. Six months after implantation, the control (PP) group showed an inflammatory response located mainly at the epithelial level (focal response), and mainly mediated by TL; however, in the experimental group, a heterogeneous chronic rejection response with scattered invasion of inflammatory cells was observed. Our results coincide with those previously reported by Ying Yao et al. with heterogenic meshes^21^.

The immunological response differed depending on the implant location (vaginal or abdominal), in line with previous reports^19^. Our study shows a greater immune response to foreign bodies in the vaginal location compared to the abdominal area in both groups; 9/18 animals studied presented an inflammatory response at the vaginal level and only 5 at the abdominal level. This different response depending on the location was more evident in the control group, where no subject had an inflammatory infiltrate at the abdominal level. The results of this study are thus consistent with observations in the real-world setting, where mesh complications are more common when the implant is placed at the vaginal level as compared with the abdominal location. The underlying mechanisms that explain this different behavior according to the location have yet to be determined, but could be influenced by the degree of host tissue vascularization.

The histological study provides information about the arrangement of collagen fibers, showing differences between the two groups. In the control group, collagen fibers were more compact, with a higher percentage of fibrosis both in the vaginal and abdominal locations, which results in a rejection response, as previously shown^19^. Along these lines, it has been described that, in PP implants, the density of collagen increases over time, unlike the inflammatory reaction that disappears over time in the abdominal location^22^. In the study group, the collagen fibers were more structured and organized, which translates into better tissue integration. This maintenance of the collagen structure was previously observed by Ying Yao et al. in heterogenic meshes^21^. In terms of biomechanical properties, this study shows that stiffness of explants diminished 6 months after implantation, especially in the vaginal location, for the study group. These results are probably due to the integration and partial degradation of the explants, predominantly in the vaginal location. These findings are in agreement with previous reports^19,23–26^.

In summary, the excellent biocompatibility of hADM grafts suggesting a good safety profile needs to be counterbalanced by the early degradation process that challenges its efficacy profile.

In terms of study limitations, the results demonstrate the safety of hADM grafts, but the animal model used does not allow us to fully evaluate graft functionality, since quadruped versus biped standing position may affect results. Another limitation of the study is the limited number of samples analyzed, as well as the size (5 × 5 mm and 5 × 10 mm) and thickness of the implants (1 mm) used. Although this size is adequate for the animal model used (rabbit), it has caused technical difficulties for the biomechanical study of the explants. In turn, the reduced thickness of the samples also makes it difficult to extrapolate the results in the human model. To better understand these limitations and comprise the potential therapeutic effect of hADM in women, our experience using samples of different thicknesses (up to 4–5 mm) and their correlation with biomechanical properties is added in the following lines. The results obtained from the preliminary in vitro studies showed that the biomechanical properties were proportional to the thickness of the sample, so that the greater the thickness, the greater the stiffness and greater resistance. However, the decision to use 1-mm matrices for this study was based on the idea of using thicknesses appropriate to the subject (in this case, small animals, thin thicknesses).

Despite the animal model limitations, the findings of our work provide a putative therapeutic alternative with relevance and applicability in the field of urogynecology.

Clinical studies in women are needed to assess the functionality of the graft. The immunologic and inflammatory response to the hADM may also be different when implanted in humans, and the biocompatibility and biomechanical properties will need to be assessed.

Unlike synthetic meshes that simply act to reinforce damaged tissue, hADM acts as a natural scaffold with full integration with the surrounding soft tissue, inducing tissue regeneration. This tentative function is compatible with the hypothesis that hADM sustains biocompatibility with the recipient tissue, and is able to abrogate the complications associated with synthetic meshes that induce local inflammation. This acellular matrix seems to act as a scaffold where the host cells can migrate and proliferate and thus regenerate the tissue where it is implanted; however, biomechanical properties may need to be improved to increase host tissue support. As the previous study conducted by our group concluded, the NZW rabbit is a suitable model for assessing materials to be used as grafts for pelvic reconstructive surgery and vaginal surgery^17^. This study provides further information on the biological properties of a new biomaterial—hADM—surgically implanted in the subcutaneous abdominal wall and in the vaginal submucosa layer of a NZW rabbit.

## Conclusions

We have investigated an alternative therapeutic option based on the use of human decellularized dermis for clinical practice in the field of urogynecology, improving current suboptimal therapies. Compared to commercial PP macroporous mesh, hADM is associated with fewer clinical complications, including vaginal mesh extrusion; as well as better incorporation into the surrounding native tissue, especially in the vaginal location. Additionally, the immunological response found in the hADM group shows a diffusely distributed cellular infiltrate, with a greater representation of B-lymphocytes. However, biomechanical analysis showed that hADM had lower resistance to stress, compared to PP mesh.

## Data Availability

All data analysed during this study are included in this published article. However, there are data collected that are not digitized, but are collected by hand in the notes of each author. Nevertheless, the datasets analysed during the current study available from the corresponding author on reasonable request.
